# Effect of dual tobacco smoking of hookah and cigarettes on semen parameters of infertile men

**DOI:** 10.18332/tid/191405

**Published:** 2024-08-05

**Authors:** Soha Albeitawi, Jehan Hamadneh, Maha Alnatsheh, Ola Soudah, Ehab Abu Marar, Laith Ayasrah, Mu’nis Alawneh, Rashed Husban, Raneem Alshraideh, Hussien Qablan

**Affiliations:** 1 Clinical Medical Sciences Department, Faculty of Medicine, Yarmouk University, Irbid, Jordan; 2 Department of Obstetrics and Gynecology, Jordan University of Science and Technology, Irbid, Jordan; 3 In Vitro Fertilization Unit, Istishari Hospital, Amman, Jordan; 4 Galway Fertility Unit, Galway, Ireland; 5 Irbid Specialty Hospital In Vitro Fertilization Center, Irbid Specialty Hospital, Irbid, Jordan

**Keywords:** hookah smoking, shisha, tobacco smoking, semen parameters

## Abstract

**INTRODUCTION:**

The research regarding the effect of hookah smoking on health is still deficient, even though it has been proven to jeopardize human health by raising the hazard of different types of cancers, infections, and cardiovascular disease. We aimed to study the effect of dual tobacco smoking (hookah and cigarettes) on semen parameters of infertile men.

**METHODS:**

In this cross-sectional study, we studied the effect of different types of smoking patterns on human semen parameters among men who visited IVF laboratories to do a seminal fluid analysis (SFA). A total number of 761 participants were included, divided into the following: 108 dual smokers, 219 hookah smokers, 222 cigarette smokers, and 212 non-smokers. To analyze the effect of dual smoking on normal morphology, an interaction term between the cigarette index and hookah index was used.

**RESULTS:**

Multivariable regression analysis after adjustment for age, BMI, education level, children, chronic diseases, varicocele, testicular surgery history, infertility duration, and cause revealed no significant difference in the sperm concentration and the percentage of progressive motility between non-smokers, cigarette smokers, or hookah smokers. However, there was a significant difference in the log of normal morphology percentage between the three groups. Cigarette and hookah smoking were significantly associated with having lower percentages of normal morphology. There was a significant difference in the log-normal morphology %, where light and heavy dual smokers had the least exponential beta of log-normal morphology %, 0.43 (95% CI: 0.33–0.55) and 0.36 (95% CI: 0.24–0.53), respectively.

**CONCLUSIONS:**

Dual tobacco smoking can adversely affect sperm morphology.

## INTRODUCTION

Tobacco smoking is considered a global health problem that connects to many morbidities and mortalities^1,2^. Cigarette smoking is well known to affect reproduction detrimentally. It is a modifiable risk factor for reproductive health.

Several studies looked at the influence of cigarette smoking on semen parameters and found that it has a notable harmful effect, especially on sperm morphology as well as motility. However, other studies found that smoking does not affect semen parameters despite the elevation in reactive oxygen species (ROS) that was detected in the seminal fluid of smokers in contrast to non-smokers among infertile men^3,4^. The results, therefore, were inconclusive.

However, smoking cigarettes has been found to have a detrimental effect on sperm motility, viability, DNA fragmentation, and semen ROS levels in fertile men, and this effect correlated with the duration and amount of smoking^5^. In a new systemic review and meta-analysis including 16 studies with 10832 infertile participants, it was found that tobacco smoking was related to a lower sperm count and a higher percentage of abnormal morphology, but motility was not affected^6^. However, in a subsequent study performed on 5146 infertile men, cigarette smoking was discovered to have a significant independent effect on sperm concentration but not on morphology and motility^7^.

Smoking is known to cause oxidative stress (OS) in spermatozoa owing to the production of reactive oxygen species (ROS)^8^. Sperm is vulnerable to oxidative stress because of the structure of the plasma membrane, which is rich in polyunsaturated fatty acids, in addition to the restricted capacity for finding and repairing DNA damage^9^.

Studies on the effect of smoking on semen parameters have had inconsistent results, where some found a significant effect on semen parameters while others failed to demonstrate any effect^10^. Very little is known about the influence of hookah smoking on human semen parameters and the male reproductive tract, particularly after long-term exposure. Moreover, it is noticed that the impact of smoking on semen parameters seems to be limited to healthy, non-infertile men^10^.

Chronic exposure to hookah smoke has been discovered to have a damaging effect on the reproductive system of rats by Ali et al.^11^ who exposed ten male mice to hookah smoke 30 min a day for six months and 11 male mice to air (control). They found that the mice exposed to hookah smoke had a notable drop in serum testosterone, estradiol, and leptin levels and a considerable rise in luteinizing hormone (LH) compared with the other group. Furthermore, there was a notable decline in the testicular antioxidants (glutathione reductase, catalase, and ascorbic acid) in the exposed group, and histopathological analysis of the testes showed a significant decline in the diameter of the seminiferous tubules with diminished spermatogenesis in the exposed mice. Transmission electron microscopy exploration demonstrated abnormal sperm structures and shapes with irregular thickening and wrinkling of the basement membranes. Another study on rats found elevated sperm DNA fragmentation and abnormal cell morphology, with an apparent decrease in sperm count, progressive motility, and serum testosterone concentration in rats exposed to hookah smoke^12^.

A recent study was performed by our team in which semen parameters were compared between hookah and non-hookah smokers and non-cigarette smokers, and no statistically vital variance between the two groups was found. However, the sample size was small (104 participants), which could have affected the results^13^. In his study, Alenzi^14^ compared the semen parameters between 100 hookah smokers and 92 non-smokers among varicocele patients. He discovered that hookah smoking has a considerable effect on sperm count (r=0.24, p<0.016) and motility (r=0.25, p=0.010)^14^.

Very few studies have compared the impact of hookah versus cigarette smoking on semen parameters. In terms of semen parameters, 30 cigarette smokers and 20 hookah smokers were compared to 50 non-smokers in men with subfertility in Saudi Arabia, and it was found that smokers had significantly lower sperm count, motility, and normal morphology than non-smokers. Still, no statistically significant differences were observed between hookah and cigarette smokers^15^. Also, in another study, the semen parameters were compared between 42 hookah smokers, 65 cigarette smokers, and 81 non-smokers from healthy men going through screening for marriage in the andrology clinic in Egypt. They found that cigarette or hookah smokers had significantly lower sperm count and lower percentage of normal morphology and motility than non-smokers. Moreover, there was no statistically significant difference in semen parameters between hookah and cigarette smokers except for normal morphology, which was less in the hookah smokers^16^. Even though the populations under investigation were different in both studies, a detrimental effect of hookah smoking on semen parameters was confirmed.

As hookah smoking is gaining popularity worldwide, it is important to investigate its effect on reproduction further. The aim of this research was to ascertain if hookah smoking has an exaggerated effect on semen parameters among dual tobacco smokers by comparing the semen parameters between dual tobacco smokers, hookah smokers only, cigarette smokers only, and non-smokers.

## METHODS

### Population and setting

At two Assisted Reproduction Technology (ART) units in Jordan, a cross-sectional study was performed on the SFA results acquired between 21 July 2020 and 21 July 2021 after obtaining an ethical approval number (IRB/2021/11) provided by the Institutional Review Board (IRB) committee at Yarmouk University and informed consent from the participants. All healthy men, smokers, and non-smokers, aged 18–45 years, who attended the andrology labs at King Abdullah University Hospital (KAUH) and Irbid Specialty Hospital for SFA testing requested by their physicians comprised the population of this study. However, men aged <18 years or aged >45 years and patients with a history of testicular trauma were excluded.

### Variables

Cigarette smoking details (cigarettes per day, duration of smoking in years, and status of passive smoking) in addition to the details about hookah smoking (type, duration, sessions per week, and duration of each session) were obtained from a questionnaire filled in by the laboratory technician, who was qualified and trained for this purpose. Moreover, reproductive and occupational history was obtained.Participants were asked if the cause of their infertility was due to a male factor, a female factor, or both, or if they were attending the fertility clinic for preimplantation genetic diagnosis (PNG). During the study period, each center performed, on average, 900 seminal fluid analyses, of which only 798 cases satisfied the inclusion criteria and agreed to participate.

### Sample preparation

Samples were gathered by ejaculation into a labeled sterile plastic canister in a room near the lab. Two to seven days of sexual abstinence were required before the day of the test. Liquefaction was allowed at a temperature of 30°C. The plastic canisters were then put in the heat stage for 30 minutes. After assessing liquefaction at 37°C in a warm stage, a disposable graduated pipette was used to measure volume and viscosity. Sperm count and motility were evaluated using a 20 magnification on a bright field microscope. Progressive motility (PR) was considered when spermatozoa moved actively linearly or in a large circle. Non-progressive (NP) motility was when all other motility patterns presented with an absence of progression, e.g. swimming in small circles or only the beat of a flagellar force could be observed. Immotile (IM) had no movement. Morphology was assessed using a bright field microscope with a magnification of 40 after mixing with an eosin/nigrosin stain. The World Health Organization (WHO) (2010) criteria were used as the reference for sperm parameters^17^. A technician performed the seminal fluid analysis in each center to avoid inter-technician inconsistency in scoring and physical properties evaluation.

### Data analysis

Participants were divided into four major groups according to their smoking status: non-smokers, dual smokers (hookah and cigarettes), hookah-only smokers, and cigarette-only smokers.

Continuous data are expressed as mean ± SD, and categorical data are expressed as frequencies and percentages. The independent sample t-test was used to find the mean differences between groups on sperm parameters, where the alpha level set at 0.05 was considered statistically significant to reject the null analysis. The cigarette smoking index was constructed from cigarette smoking duration in years multiplied by the daily number of cigarettes. Participants were then categorized as non-cigarette smokers, light smokers, moderate smokers, and heavy smokers, and based on the sample distribution quartiles: Q1–2 light, Q3 moderate, and Q4 heavy smokers. The hookah smoking index was constructed from the duration in years multiplied by the weekly number of hookahs. Participants were then categorized as non-cigarette smokers, light smokers, moderate smokers, and heavy smokers, and based on the sample distribution quartiles: Q1–2 light, Q3 moderate, and Q4 heavy smokers. Bivariate analysis was done using chi-squared for categorical variables and ANOVA for continuous variables. For regression modeling, all dependent variables were log-transformed to adjust for the highly right-skewed nature of the semen counts. A linear regression model was used to test the hypothesis, adjusting for demographic variables and potential confounders. Results are reported as the exponential of the beta coefficient for the model involving log-transformed outcomes; the exponentiation of the coefficients converts them back to the original scale, transforming them from log differences to multiplicative effects (i.e. percentage changes). An alpha level set of 0.05 was considered statistically significant to reject the null analysis. All analyses were performed using SPSS v.24, Armonk, NY (IBM Corp).

## RESULTS

The total number of participants was 798, with 37 being excluded, 35 having incomplete data, and 2 being only electronic cigarette smokers. Therefore, the total number included in the study was 761 participants divided into the following: 108 dual smokers, 219 hookah smokers, 222 cigarette smokers, and 212 non-smokers. Baseline characteristics areshown in Table 1. The average age of the participants was 35.4 years (SD=7.69), with an average BMI of 28.4 kg/m^2^ (Table 1). The majority of participants reported having no chronic diseases, varicocele, or history of testicular surgery, with the prevalence of these conditions being 9.7%, 19.3%, and 12.9%, respectively. The primary cause of infertility was unexplained (31.4%), followed by female factors (30.6%). Infertility duration was <5 years for 63.5% of the participants while only 9.6% had been infertile for >10 years. Approximately 70.1% of the participants were smokers, with 29.2% smoking cigarettes, 28.8% using shisha, and 12.9% using both.

**Table 1:** Baseline sample characteristics surveyed among infertile men, smokers, and non-smokers, aged 18–45 years, attending ART Unit between July 2020 and July 2021 in Jordan (N=761)

*Characteristics*	*n*	*%*
**Age** (years), mean (SD)	35.4 (7.69)	
**BMI** (kg/m^2^), mean (SD)	28.4 (6.4)	
**Education level**		
<Bachelor’s	304	39.9
Bachelor’s	387	50.9
>Bachelor’s	70	9.2
**Have children**	289	38
**Chronic illness**	74	9.7
**Infertility duration** ( years )		
0–5	483	63.5
6–10	205	26.9
11–15	42	5.5
>15	31	4.1
**Infertility cause**		
Female	233	30.6
Male	176	23.1
Mixed	99	13
PGD	14	1.8
Unexplained	239	31.4
Varicocele	147	19.3
History of testicular surgery	98	12.9
**Smoking status**		
Non-smoker	212	27.9
Cigarettes only	222	29.2
Shisha only	219	28.8
Dual smoker	108	14.2
**Cigarette smoking index**		
None	431	56.6
Light	62	8.1
Moderate	76	10
Heavy	192	25.2
**Shisha smoking index**		
None	434	57
Light	173	22.7
Moderate	56	7.4
Heavy	98	12.9

Table 2 demonstrates the smoking details, while Table 3 illustrates the bivariate analysis of the smoking status. There was a statistically significant difference in sperm concentration, progressive motility percentage, and morphologically normal spermatozoa percentage. Dual tobacco smokers and hookah smokers had the lowest percentage of progressively motile sperms and morphologically normal sperms. On the other hand, non-smokers had the lowest sperm concentration but the highest percentage of morphologically normal sperm.

**Table 2:** Smoking details, surveyed among infertile men, smokers, and non-smokers, aged 18–45 years, attending ART Unit between July 2020 and July 2021 in Jordan (N=761)

*Characteristics*	*Categories*	*n*	*%*
**Smoking type**	None	212	27.9
	Cigarette	222	29.2
	Shisha	219	28.8
	Dual	108	14.2
**Cigarette smoking status**	No	431	56.6
	Yes	330	43.4
**Cigarette smoking duration** (years)	None	431	56.6
	0–5	28	3.7
	6–10	63	8.3
	11–20	175	23.0
	>20	64	8.4
**Number of cigarettes per day**	None	431	56.6
	0–5	28	3.7
	6–10	60	7.9
	11–20	129	17.0
	>20	113	14.8
**Cigarette smoking index**	None	431	56.6
	Light	62	8.1
	Moderate	76	10.0
	Heavy	192	25.2
**Shisha smoking status**	No	434	57.0
	Yes	327	43.0
**Shisha smoking duration (years)**	None	434	57.0
	0–5	101	13.3
	6–10	129	17.0
	11–20	84	11.0
	>20	13	1.7
**Shisha smoking frequency (number per week)**	None	435	57.2
	1–3	135	17.7
	3–6	59	7.8
	≥7	132	17.3
**Shisha smoking index**	None	434	57.0
	Light	173	22.7
	Moderate	56	7.4
	Heavy	98	12.9

**Table 3:** Bivariate analysis for outcome variables semen counts, progressive motility, and normal morphology, surveyed among infertile men, smokers, and non-smokers, aged 18–45 years, attending ART Unit between July 2020 and July 2021 in Jordan (N=761)

*Baseline sample characteristics*	*Semen counts % mean (SD)*	*Progressive motility % mean (SD)*	*Normal morphology % mean (SD)*
**Age** (years), β	-0.58	-0.05	-0.06
**BMI** (kg/m^2^), β	-0.03	0.02	-0.01
**Education level**			
<Bachelor’s	27.64 (2.07)*	20.39 (1.1)*	3.3 (0.41)*
Bachelor’s	33.25 (1.8)	21.6 (1.0)	4.6 (0.35)
>Bachelor’s	37.58 (4.27)	28.8 (2.3)	5.4 (0.83)
**Have children**			
No	28.36 (1.65)*	19.2 (0.9)*	3.67 (0.3)*
Yes	36.04 (2.1)	25.99 (1.15)	4.94 (0.41)
**Chronic illness**			
No	32.47 (1.37)*	22.5 (0.75)*	4.3 (0.26)
Yes	21.98 (4.15)	15.4 (2.3)	3.2 (0.82)
**Infertility duration** (years)			
0–5	35.75 (1.6)*	24.4 (0.88)*	4.7 (0.32)*
6–10	26.25 (2.5)	19.64 (1.63)	3.6 (0.49)
11–15	18.11 (5.5)	13.61 (3.01)	1.92 (1.08)
>15	14.5 (6.7)	6.92 (3.47)	1.6 (1.2)
**Infertility cause**			
Female	40.99 (2.2)*	28.3 (1.2)*	6.9 (0.4)*
Male	12.14 (2.56)	10.9 (1.4)	1.8 (0.5)
Mixed	21.84 (3.4)	16.17 (1.87)	1.9 (0.7)
PGD	43.36 (9.04)	27.64 (4.95)	3.1 (1.8)
Unexplained	39.52 (2.2)	25.3 (1.2)	4.04 (0.43)
**Varicocele**			
No	33.57 (1.44)*	23.2 (0.8)*	4.2 (0.28)
Yes	22.46 (2.96)	16.82 (1.6)	3.99 (0.6)
**History of testicular surgery**			
No	33.93 (1.37)*	23.35 (0.76)*	4.3 (0.27)
Yes	14.13 (3.62)	11.34 (1.96)	2.9 (0.72)
**Cigarette smoking index**			
None	31.16 (1.74)	22.9 (0.96)	5.4 (0.33)*
Light	28.48 (4.56)	21.12 (2.5)	2.34 (0.88)
Moderate	36.05 (414)	19.26 (2.27)	2.5 (0.78)
Heavy	31.23 (2.6)	20.54 (1.42)	2.7 (0.49)
**Shisha smoking index**			
None	28.06 (1.7)*	19.86 (0.94)*	4.7 (0.33)*
Light	35.37 (2.7)	21.4 (1.5)	2.7 (0.53)
Moderate	37.43 (4.8)	27.5 (2.6)	3.08 (0.9)
Heavy	35.9 (3.6)	27.9 (1.98)	4.36 (0.7)

* p<0.05, there is a statistically significant difference between groups.

In the bivariate analysis, all outcome variables showed significant differences based on education level, infertility duration, and causes of infertility (Table 2). As the duration of infertility increases, semen parameters (count, progressive motility, and normal morphology) decrease in a dose-response manner. Additionally, semen parameters were the lowest for male causes of infertility and highest for female causes. Factors such as not having children, having a chronic illness, and a history of varicocele and testicular surgery were associated with poorer semen parameters. Neither cigarette smoking nor shisha smoking was associated with semen counts and progressive motility (p>0.05). However, shisha smoking significantly affected normal morphology compared to non-shisha smokers. Light shisha users had an average normal semen morphology of 2.7 (SD=0.53), compared to non-shisha users at 4.7 (SD=0.33) (p<0.05). Moderate and heavy shisha smokers had slightly higher average normal morphology compared to light users, yet their values were still significantly lower than those of non-shisha users, at 3.08 and 4.36, respectively (Table 3).

### Cigarette smoking and shisha smoking versus non-smoking

In the multivariable regression analysis, after adjusting for age, BMI, education level, children, chronic diseases, varicocele, history of testicular surgery, infertility duration, and cause, there were no significant differences in semen counts and the percentage of progressive motility between cigarette smokers and non-smokers. Similarly, shisha smoking was not significantly associated with semen counts and progressive motility. However, both cigarette and shisha smoking were significantly associated with lower normal morphology. The exponential beta for the regression coefficient for light cigarette smokers and the log-normal morphology percentage was 0.66 (95% CI: 0.56–0.77), indicating that light cigarette smoking is associated with a 34% decrease in normal morphology. Moderate and heavy cigarette smokers also exhibited decreased normal morphology, with exponential betas of 0.63 (95% CI: 0.54–0.73) and 0.68 (95% CI: 0.62–0.76), respectively. Shisha smoking similarly resulted in a decrease in normal morphology; light shisha smokers had an exponential beta of 0.66 (95% CI: 0.60–0.74), moderate shisha smokers had 0.80 (95% CI: 0.67–0.94), and heavy shisha smokers had 0.81 (95% CI: 0.70–0.92).

### Dual smoking versus non-smoking

To examine the effects of dual smoking on normal morphology, an interaction term between cigarette and shisha indices was used (Table 4). The exponential beta for the interaction terms indicates a more pronounced decrease in normal morphology for dual smokers compared to those who smoked only cigarettes or only shisha. For example, light dual smokers had an exponential beta of 0.43 (95% CI: 0.33–0.55), reflecting a 57% decrease in normal morphology compared to non-smokers. This is more significant than the 34% decrease observed for light cigarette smokers only (0.66; 95% CI: 0.56–0.77).

**Table 4:** Multivariable regression analysis* to test the cigarette and shisha smoking impact on semen parameters, surveyed among infertile men, smokers, and non-smokers, aged 18–45 years, attending ART Unit between July 2020 and July 2021 in Jordan (N=761)

*Parameter*	*Log (semen count %)*		*Log (progressive motility %)*		*Log (normal morphology %)*	
	*Exp(β) (95% CI)*	*p*	*Exp(β) (95% CI)*	*p*	*Exp(β) (95 % CI )*	*p*
**Cigarette index**						
None	1		1		1	
Light	1.01 (0.83–1.23)	0.90	0.98 (0.8–1.19)	0.80	0.66 (0.56–0.77)	<0.001
Moderate	1.20 (1.0–1.44)	0.05	0.83 (0.7–1.0)	0.05	0.63 (0.54–0.73)	<0.001
Heavy	1.07 (0.94–1.22)	0.30	0.90 (0.79–1.02)	0.10	0.68 (0.62–0.76)	<0.001
**Shisha index**						
None	1		1		1	
Light	1.12(0.98–1.28)	0.08	0.89 (0.78–1.01)	0.07	0.66 (0.6–0.74)	<0.001
Moderate	1.22 (1.0–1.5)	0.06	1.06 (0.87–1.31)	0.60	0.80 (0.67–0.94)	0.01
Heavy	1.14 (0.96–1.35)	0.13	1.08 (0.92–1.28)	0.30	0.81 (0.7–0.92)	0.00
**Dual cigarette and shisha smoking effect** (interaction term)						
(Cigarette index=None) × (Shisha index=None)					1	
(Cigarette index=Light) × (Shisha index=Light)					0.43 (0.33–0.55)	<0.001
(Cigarette index=Moderate) × (Shisha index=Moderate)					0.70 (0.43–1.16)	0.17
(Cigarette index=Heavy) × (Shisha index=Heavy)					0.36 (0.24–0.53)	<0.001

* All models were adjusted for age, BMI, education level, children, chronic diseases, varicocele, testicular surgery history, infertility duration, and cause. Linear model with log-transformation was used to calculate p-value.

To examine the effect of dual smoking on normal morphology, an interaction term between the cigarette index and hookah index was used (Table 4). There was a significant difference in the log-normal morphology %, where light and heavy dual smokers had the least exponential beta of log-normal morphology %, 0.43 (95% CI: 0.33–0.55) and 0.36 (95% CI: 0.24–0.53), respectively.

## DISCUSSION

Smoking impairs spermatogenesis and sperm maturation and comprises the function of spermatozoa^4^. It has been reported that nicotine causes Leydig cell apoptosis and inhibition of androgen synthesis, leading to male reproductive hormone impairment^18^. Lead and other toxins exist in tobacco, which directly mutilate the process of spermatogenesis and sperm function^19^. Nicotine has a considerable effect on sperm count and morphology^20^. This means that smokers are expected to have less sperm concentration than non-smokers.

Nevertheless, in the initial bivariate analysis, we found that non-smokers had the lowest sperm concentration. This could partially be explained by the fact that the population of the study is infertile due to other possible hidden pathologies. Moreover, there could be an effect of confounding factors such as chronic illnesses, duration of infertility, and history of testicular surgery. These have been proven to affect the results since after adjustment of the confounding factors using the multivariable regression analysis, there was no significant difference in sperm concentration between non-smokers and smokers.

Moreover, sperm motility was found to be negatively correlated to seminal plasma cotinine levels, but a meta-analysis on the effect of smoking on the semen of an infertile population^6^ did not confirm this. This coincides with our results, which found no significant difference in sperm progressive motility between non-smokers and smokers in all categories.

It has been demonstrated that hookah tobacco has a higher concentration of lead (0.83 mg/kg) in comparison to cigarette tobacco (0.19 mg/kg)^21^. Furthermore, nicotine and cotinine levels were found to be elevated in serum, urine, and saliva after hookah smoking, and seminal plasma cotinine levels are related to serum levels. The levels in the seminal plasma were significantly higher than in serum^22,23^. Therefore, hookah smoking is expected to have a tremendously damaging effect on semen parameters. The harmful effect of hookah smoking on sperm count, motility, and morphology has been demonstrated in animal studies. The proposed mechanism could be secondary to the suppressive effect on testosterone and oxidative stress (OS), as lower testosterone levels and high DNA fragmentation were observed in exposed rats^11,12^.

Moreover, smoking is known to be one of the causative factors of OS, resulting from excessive levels of ROS coupled with a deficiency in antioxidants. The active transfer of cigarette components through the blood-testis barrier is responsible for oxidative stress-induced damage^24,25^. Tobacco contains nicotine, cadmium, and lead, which are mutagenic and carcinogenic and cause damage to cells undergoing rapid multiplication, including germ cells in the testis^26^.

Our results are comparable with the literature in terms of morphology, which has been confirmed to be adversely affected by tobacco smoking. Non-smokers had a higher percentage of normal morphology, and dual tobacco smokers had the worse morphology, as we found in the initial bivariate analysis. However, hookah smokers had a better normal morphology percentage than cigarette smokers, which contradicts what is expected as hookahs are known to have higher concentration of toxins and to expose their user during a 1-hour session, to 100–200 times the volume of smoke inhaled in one cigarette^27^. This could be explained by the higher combustion temperature in cigarette smoking, which produces a large variety of toxic compounds. Still, the low temperature of hookah smoking and added sugary flavorings and glycerol results in particles that are less concentrated in many toxic compounds^28^. Moreover, though we asked about the average duration of a hookah session, we do not know how many puffs are inhaled in each session and the depth of an inhalation in each puff, which would be difficult to assess and therefore may mask any possible effect of hookah smoking. However, dual smokers had the worst percentage of normal morphology of sperms, which may indicate a synergistic damaging effect of a hookah on top of cigarette smoking.

Concerning sperm progressive motility, our results coincide with the latest meta-analysis that smoking does not affect sperm motility, as was evident in the multivariable regression analysis. However, in an early study conducted in Saudi Arabia among 68 infertile men and 29 fertile men, smoking was found to improve motility among the infertile men significantly^29^.

When exploring the effect on sperm concentrations, our results are in contrast to what was evident in the last meta-analysis, where we found that non-smokers had the lowest sperm concentration. Still, hookah smokers had the highest concentration, followed by dual smokers and then cigarette smokers. Although this effect was not evident in the multivariable regression analysis after adjustment for the different variables, this could be due to different populations from different demographic areas and probably environmental factors, as most of the participants included in the meta-analysis were from regions that are different from our population. Moreover, since the participants were infertile, there might be other hidden factors responsible for their infertility that affected their sperm count. It is crucial to bear in mind that smoking is widely spread in Jordan. It has been estimated that nearly 50% of men in Jordan are current smokers, and the highest prevalence (63%) is among men aged 25–34 years. There are no strictly applied regulations to prohibit smoking even in workplaces, so there is a high probability that non-smokers could be passive smokers^30^. Therefore, this could act as a confounding factor. In addition, whether the non-smokers are ex-smokers or not, could not be ascertained as we did not ask about their history of smoking.

### Strengths and limitations

As it is difficult to recruit healthy men for semen parameters study, most of the studies were conducted on men attending infertility clinics in which they could have infertility, whether secondary to a male or female factor. This would create selection bias and could be one reason for inconsistent results. These patients might have other hidden pathologies that would modify the effect of smoking diversely from healthy, fertile men. Moreover, the difficulty of quantifying the exposure to hookahs is a strong limitation of such a study, as it is extremely challenging to determine the amount of smoke inhaled, the depth of inhalation, and the type of hookah being smoked whether it is flavored or not. Moreover, there is no fixed concentration of the hookah constituents, which could affect the amount of harmful substances present. In addition, there is the possibility of passive exposure in non-smokers, which we did not inquire into. Furthermore, such a study design could not elicit a causal relationship.

Our study is characterized as being unique in confronting dual tobacco smoking on semen parameters. We excluded patients with a previous history of testicular trauma to avoid any possible harmful effects from other factors other than smoking. Moreover, we designed the smoking index for hookah smoking and dual tobacco smoking based on the smoking index of cigarette smoking.

To study the impact of hookah smoking and to quantify its effect is difficult. The process of hookah smoking is variant and inconsistent, which will affect the amount of inhaled smoke and toxins. Accordingly, studies may end up with variable results. Therefore, it is essential to measure the levels of cotinine and toxins in serum and seminal plasma, compare their levels between different types of smokers and non-smokers, and then correlate them with semen parameters. Moreover, to avoid the effect of pathologies underlying infertility itself, semen parameter studies on healthy, fertile men are urgently needed.

## CONCLUSIONS

Dual tobacco smoking can be associated with a detrimental effect on sperm. However, further studies are needed to explore the effect of dual tobacco smoking on male fertility.

## Data Availability

The data supporting this research are available from the authors on reasonable request.
